# Molecular Dynamics Insights into Substrate-Induced Gradient Stiffness and Vibrational Modes in P3AT Thin Films

**DOI:** 10.3390/ma19143044

**Published:** 2026-07-15

**Authors:** Peng Wan, Wenzhan Zhang, Hongji Yuan, Xianwei Xu

**Affiliations:** 1 School of Aeronautics and Astronautics Engineering, Nanchang Institute of Technology, Nanchang 330044, China; 2 School of Electronics and Information Engineering, Nanchang Institute of Technology, Nanchang 330044, China; jawenzhan@163.com

**Keywords:** P3AT, gradient stiffness, phonon vibrational modes, energy decomposition

## Abstract

In this study, we reveal the emergence of a tri-regime gradient in stiffness across substrate-supported poly(3-alkylthiophene) (P3AT) thin films, comprising an adsorbed region, a bulk-like region, and a free surface region. The stiffness distribution is found to be largely independent of the degree of polymerization but is significantly modulated by side chain length and temperature. Specifically, longer side chains (bead count = 4) expand the adsorbed and free surface regions, while elevating temperature above the glass transition leads to an order-of-magnitude reduction in stiffness. Phonon mode analysis demonstrates a clear inverse correlation between vibrational frequency and both the degree of polymerization and temperature, with side chain length exerting minimal influence. A high phonon mode similarity index between the main and side chains indicates coupled vibrational dynamics. Interfacial energy decomposition confirms that van der Waals interactions, particularly through distinct π–π stacking, dominate the substrate adhesion. These findings provide fundamental insights into the nanoscale thermomechanical properties of P3AT thin films on silica substrates, offering valuable guidance for the interface engineering of P3AT-on-silica systems in organic electronics.

## 1. Introduction

Conjugated polymers belong to functional organic materials [1] and have been used in many organic optoelectronic devices [2,3,4], organic field-effect transistors [5,6], photocatalysis, and bioelectronics [7], and polymer solar cells [8,9,10]. Among them, poly(3-alkylthiophenes) (P3ATs) has received numerous studies due to its unique characteristics [6,11]. As the alkylated polythiophene and a semiconductive polymer [12] (polythiophene polymers can conduct electricity because they have π electrons in the thiophene ring), it has great importance in the fields of electronics and optoelectronics. At present, there is little research on the behavior of P3AT at the interface, and most of them focus on experiments or on linear homopolymer chains without side chains, using molecular dynamics simulations and other interfacial and surface properties [13,14,15,16,17,18]. The broad promise of polythiophene-based systems for functional materials has been extensively documented. For instance, polythiophenes and their derivatives have been employed as active layers in organic field-effect transistors, as donor materials in bulk-heterojunction solar cells, and as conductive components in organic thermoelectrics, owing to their tunable optoelectronic properties and solution processability [19]. Moreover, in polymer-based composites, the interfacial adhesion and mechanical matching between the conjugated polymer and the inorganic filler or substrate play a decisive role in determining the overall device performance and long-term stability [20]. Despite this progress, the nanoscale interfacial behavior of P3AT thin films, including substrate-induced stiffness gradients and the coupling between main chain and side chain dynamics, remains poorly understood. Elucidating these interfacial characteristics at the molecular level is essential for the rational design of P3AT-based organic electronic devices.

In simulations, the thin film is divided into several regions. According to the rules of Zhou et al. [21] and Tian et al. [22], it is divided into three regions: the adsorbed layer, the bulk region, and the gradient region or interface region. Zhou et al. [21] studied the dynamic propagation depth of substrate effect in polymer film. The L-J units they employed are dimensionless. They investigated the effects of substrate attraction strength and polymer chain length (degree of polymerization) on diffusion and conformation of adsorbed and non-adsorbed chains on the substrate-supported thin film. Their results show that adsorbed chains’ average conformation properties are weakly dependent on substrate attraction strength. The adsorption region is composed of two types of conformations: strongly adsorbed chains with a flat compressed conformation(trains) and weakly adsorbed tails. These simulation results demonstrate a strong correspondence with experimental findings [22,23]. Tian et al. [22] conducted experimental investigations on metastable polymer adsorption at the interface. The results show that there is a dynamical gradient region between the bulk and adsorbed regions at the metastable state. The extension of loosely adsorbed chains into the film interior can promote the propagation of interface-derived suppressed dynamics. This conclusion is similar to some research [24,25,26], which studied the conformation of loops. Zuo et al. [25] investigated the flattened adsorbed chains with larger loops through an experiment. Their results show that by modulating the dimensions of the loop, highly efficient propagation of suppressed interfacial dynamics can be achieved. Gao et al. [26] investigated the influence of loop length (degree of polymerization) and rigidity on other linear chains in thin films by anchoring the loop structure onto the substrate; an irreversible adsorption was set between the loop chain and solid substrate. This setting is called confined molecular dynamics. The results show that increasing the length of loop chains can more effectively confine the motion of other linear chains, and there is an optimal loop chain rigidity that strongly confines the motion of linear chains. Lu et al. [24] studied the effect of attraction strength of substrate, polymer concentration, and chain length on the conformation and stability of adsorbed homopolymer chains by Langevin dynamics. Their results show that loop size and adsorption stability increase with increasing chain length, while inter-polymer interactions weaken the effect of substrate attraction. Similar to Gao’s setting, they also used dimensionless L-J units. Their results show that loop size and adsorption stability increase with increasing chain length, and the inter-polymer interactions weaken the effect of substrate attraction. Those findings have substantial implications for surface modification.

In addition to interfacial conformation, the mechanical properties of thin films have also garnered significant attention [13,23,27,28,29,30,31]. Xia et al. [29] studied the local stiffness of a free-standing polymer thin film using coarse-grained molecular dynamics. They investigated the Debye–Waller factor of three polymers: polystyrene, poly (methyl methacrylate), and poly (1-ethylcyclopentyl methacrylate). Their results show that the local stiffness decays almost linearly near the free surface. In the interior region, the local stiffness increases and then becomes nearly independent of the film position. They also evaluated the elastic moduli. Their research holds valuable implications for our study. Currently, a majority of the research focuses on linear chains, with relatively fewer studies conducted on polymers with side chains. Hsu et al. [27] studied the side chain dynamics in thin films and *T*_g_ differences caused by the free surface effects of PS and PMMA. They found that increasing the mass fraction of the side chain relative to the repeat unit in coarse-grained models amplifies side chain fluctuations and suppresses the free-surface effect on *T_g_*. Zhang et al. [32] investigated the influence of side chain length on the main chain phase structure and side chain ordering of P3AT thin films through experimental research. They found that the outer side chain carbon’s mobility is higher than that of the inner carbon close to the backbone. Research on the dynamics of side chains in P3AT using coarse-grained molecular dynamics is currently lacking.

Coarse-grained molecular dynamics has the advantages of accelerating kinetic implementation and shortening simulation time and is often applied in the field of polymer simulation [14]. Schwarz et al. [33] developed a poly(3-hexylthiophene) coarse-grained model and studied its solution-phase self-assembly. Wang et al. [34] used an energy-renormalization approach to establish a temperature-transferable coarse-grained model of poly(3-hexylthiophene). They adopted the bottom-up method called the iterative Boltzmann inversion method to extract the bonded and non-bonded parameters. Their results exhibit a high degree of simulation effectiveness. Alessandri et al. [35] adopted a coarse-grained method to simulate the large-scale morphological organization of a mixture, which is composed of poly(3-hexylthiophene) and phenyl-C61-butyric acid methyl ester (PCBM). Nair et al. [36] used another coarse-grained technique called dissipative particle dynamics to study the structural stability and interfacial behavior of polymer-grafted nanoparticles. Inspired by the above literature, we investigated the adsorption behavior of poly(3-alkylthiophene) at the silica interface through coarse-grained molecular dynamics.

The present work adopted coarse-grained molecular dynamics to study the conformation and adsorption of P3AT on a silica substrate. We studied the conformation of polymer chains adsorbed on the substrate. Next, we used the Debye–Waller factor [29,30] to quantify the local stiffness distribution of polymers on the substrate at different polymerization degrees, side chain lengths and temperatures. In addition, the phonon density of state was calculated to describe the vibrational modes under these three conditions. Finally, we discussed the side chain dynamics by using the similarity index and implemented an energy decomposition for the interfacial interaction.

## 2. Simulation Methods

**Figure 1:**
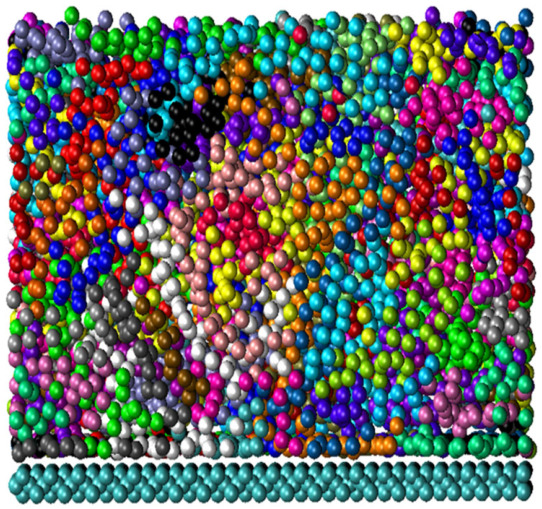
Schematic diagram of P3AT thin film on silica. Each P3AT chain is rendered in a different color for visual distinction only, with no physical meaning. The cyan region at the bottom represents the silica substrate, above which the P3AT polymer chains are stacked.

The coarse-grained molecular dynamics simulation was performed by using LAMMPS [37] in this article. Simulations are carried out in a cubic box with 100 Å × 100 Å × h, where h depends on the density of P3AT. Polymer chains are stacked on the surface of a silica substrate to form a thin film, as shown in Figure 1. Each P3AT chain in the figure is represented by a different color, and the lower cyan part represents the silicon dioxide substrate.

Periodic boundary conditions (PBC) are used in three directions. The energy minimization method used was the conjugated gradient algorithm before the dynamics run. The system was subjected to three annealing cycles: each cycle started at 800 K and was cooled stepwise to 100 K at a rate of 10 K/ns, with a 1ns equilibration stage at every 50 K interval. After the final cycle, the system was further equilibrated at the target temperature for 2.5 ns. To achieve the predetermined density value of the polymer, the upper and lower surfaces of P3AT are limited by two silica plates. After sufficient annealing treatment, the upper plate was removed. During the annealing, a pure repulsive force is set between the polymer and silica surface. Equilibration of the thin-film system was monitored through the time evolution of multiple observables, including the total potential energy, overall film thickness, number of adsorbed beads in direct contact with the substrate, and layer-resolved density profiles. All monitored quantities were observed to stabilize after the first 2 ns of the production run, fluctuating around steady mean values with no systematic drift. Accordingly, the initial 2 ns were discarded as the equilibration phase, and all subsequent analyses were performed exclusively on the equilibrated portion of the trajectory (*t* > 2 ns).

The nonbonded interactions between coarse-grained beads are described by the standard Lennard-Jones (L-J) 12-6 potential. The bead diameter is approximately 4.0 Å (based on *σ* ≈ 3.6Å), and the nonbonded cutoff was set to 1.2 nm. Complete L-J parameters and bonded interaction potentials for the P3AT model were taken from the temperature-transferable coarse-grained force field developed by Wang et al. [34], to which the reader is referred for full details. Their parameters are obtained through energy renormalization [34] and have temperature transferability. The bond interaction potential functions, such as bond length, bond angle, and dihedral angle, were extracted from the all-atom simulation using the iterative Boltzmann inversion method. The model has fully retained the rigid characteristics of the main chain thiophene ring and the flexible degrees of freedom of the side chain alkyl and has been independently verified by the experimental density and glass transition temperature. Therefore, the flexibility and local chemical structure of the chain have been accurately inherited in the mapping process from all atoms to coarsening, and there is no need to repeat the fitting at the coarsening level with the radius of gyration or duration as the goal.

**Figure 2:**
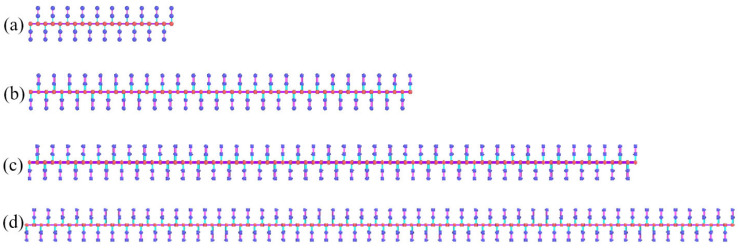
P3AT chains with different degrees of polymerization: (**a**) 20, (**b**) 50, (**c**) 80, and (**d**) 100 monomers. The total number of coarse-grained beads was kept constant (2500 beads) across all systems to ensure a consistent basis for comparison of film thickness and density profiles.

When studying the impact of polymerization degree, the polymerization degrees of P3ATs are 20, 50, 80, and 100, respectively, as shown in Figure 2. In our model, the total number of beads was kept constant (2500 beads) across different DPs; therefore, the number of chains decreases as DP increases. Specifically, polymer chains with DP = 20, 50, 80, and 100 have 125, 50, 32, and 25 chains, respectively. The number of beads in the polymer was kept the same under different polymerization degrees. The chosen range of DP (20–100) covers the transition from short oligomers to polymer chains with molecular weights relevant to experimental P3AT systems (M_n_ ≈ 20–30 kDa for DP = 100). As shown in Section 3.2, the local stiffness and vibrational frequencies become essentially saturated for DP ≥ 50, confirming that this range is sufficient to capture the full chain-length dependence without the need for higher polymerization degrees. The film was divided into 20 layers of equal thickness along the direction normal to the substrate, yielding a per-layer thickness of approximately 3.61 Å. This value is comparable to the coarse-grained bead diameter (*σ* ≈ 4 Å), allowing adequate spatial resolution of the interfacial gradient while maintaining reasonable statistical sampling within each layer [21,29].

**Figure 3:**
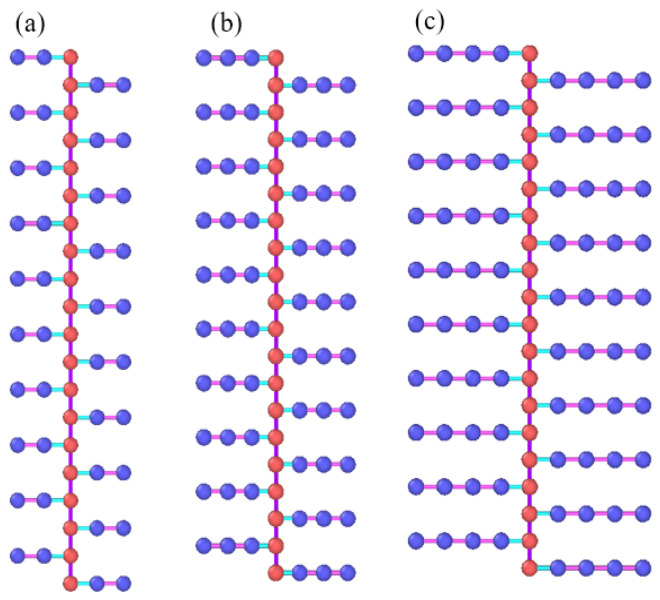
P3AT polymers with (**a**) 2 beads, (**b**) 3 beads, (**c**) 4 beads at polymerization degree = 20; the blue beads represent the side chain alkane, red beads represent the main chain backbone.

The side chain bead numbers in coarse-grained P3AT are 2, 3, and 4, respectively, as shown in Figure 3. Polymer chains with the number of side chain beads 2, 3, and 4 have 125, 94, and 75 chains, respectively. The schematic diagrams shown in Figure 2 are at different degrees of polymerization, side chain bead numbers, and temperatures. We discussed the stiffness, vibrational modes, main chain–side chain similarity index, and energy decomposition. The model with beads and springs was created using our own Python code, and molecular dynamics trajectories were analyzed with a practical object-oriented Python toolkit, MDAnalysis (version 2.7.0) [38]. OVITO (version 3.10.6) [39] and VMD (version 1.9.4) [40] were used for the simulation results. To estimate the statistical uncertainty of the reported quantities, the equilibrated production trajectory (*t* > 2 ns) of each system was divided into five blocks of equal duration. The mean value of each observable was computed within each block, and the standard deviation across the five block averages was used as the uncertainty estimate. For key observables such as the Debye–Waller factor and phonon frequencies, the block-averaged standard deviations are explicitly reported in the text and figure captions. Although independent replica simulations were not performed due to computational constraints, the parameter scan over multiple chain lengths, side chain lengths, and temperatures provides an internal consistency check, as systematic trends far exceeding the block-averaged fluctuations are observed in all cases.

## 3. Results and Discussion

### 3.1. The L-J Parameters and Adsorption Conformation

**Figure 4:**
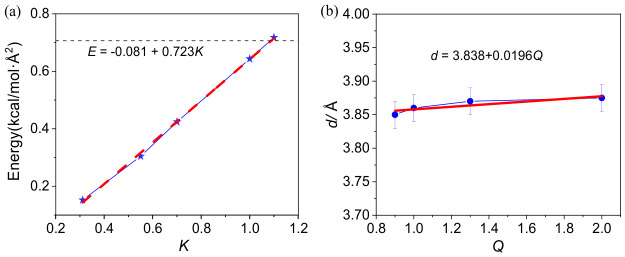
Determination of the scaling parameters *K* and *Q*. (**a**) Binding energy *E* as a function of *K*. The horizontal dashed line marks the all-atom target value of 0.7068 kcal/(mol·Å^2^). (**b**) Binding energy *E* and equilibrium distance *d* (in Å) as functions of *Q*. The equilibrium distance d refers to the bead-substrate separation.

To find the L-J parameters between the different beads, we introduced the scaling parameters *K* and *Q*, respectively. *K* is the scaling factor of the energy parameter *ε* in the coarse-grained Lennard-Jones potential, which is defined as $\varepsilon_{\mathrm{CG}}=K\varepsilon_{\mathrm{AA}}$. Its physical meaning is to adjust the strength of the non-bonded interaction between coarse-grained beads, which directly affects the size of the interface binding energy. *Q* is the scaling factor of the distance parameter *σ*, defined as $\sigma_{\mathrm{CG}}=Q\sigma_{\mathrm{AA}}$. Its physical meaning is to adjust the effective size of the beads and then control the equilibrium distance between beads and between beads and the substrate. Different values of *K* and *Q* can produce different equilibrium distances and binding energy [41,42]. According to our all-atom calculations, the target binding energy is *E* = 0.7068 kcal/(mol·Å^2^) and the equilibrium distance is about 3.52 Å. We then adjusted the scaling parameter *K* in the coarse-grained model to match the binding energy and obtained *K* = 1.089 (Figure 4a). Further, fixing *K* = 1.089 and adjusting *Q*, it is found that the equilibrium distance *d* is insensitive to the change of *Q*, and *Q* = 1.006 can maintain the equilibrium distance consistent with the results of all atoms (Figure 4b). This fitting logic is to lock the interface energy intensity with *K* first and then confirm that the influence of *Q* on the distance can be ignored, so as to determine the non-bond parameters based on the interface thermodynamics, rather than the force field, by fitting the chain conformation parameters.

As for the fitting process, under the condition of fixed *Q* equal to 1, we calculated the variation of binding energy with *K*, and found that when *K* increased to 1.089, the binding energy given by the coarsening model just passed through the target value, so *K* = 1.089 was determined as the best energy scaling factor. Then, we fixed *K* = 1.089 and investigated the changes of binding energy and equilibrium distance with *Q*. The results show that the variation of equilibrium distance is less than 0.03Å and the fluctuation range of binding energy is less than 0.05 kcal/(mol·Å^2^) in the range of *Q* from 0.98 to 1.02, and the dependence of both on *Q* is weak. This analysis shows that the fine adjustment of the distance parameter *σ* has almost no measurable effect on the thermodynamic quantities of the whole interface, so taking *Q* = 1.006 is numerically equivalent to *Q* = 1, which fully meets the consistency with the results of all atoms.

**Figure 5:**
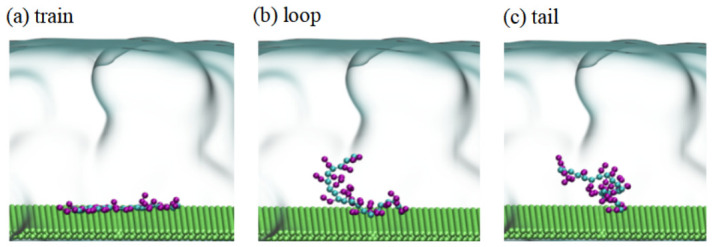
Schematic diagram of train (**a**), loop (**b**) and tail (**c**) conformations.

In the coarse-grained simulation, the substrate is modeled as a rigid repulsive wall with fixed position, and the non-bond repulsion between the beads and the substrate is controlled by $\sigma_{\mathrm{CG}}$. When *Q* is slightly disturbed near 1, the displacement of the L-J repulsive wall is far less than the root mean square displacement of the bead under thermal vibration at room temperature, so the apparent equilibrium distance is essentially determined by a relatively wide flat area of the potential well and is insensitive to small changes in *σ*. The conformational properties of nano-confined polymers depend primarily on the strength of the substrate. The interface adsorption region is composed of flat trains, loose loops, and tails, with three typical conformations shown in Figure 5. In our analysis, a train is defined as a continuous segment in which three or more consecutive beads along the chain are in direct contact with the substrate (bead-substrate distance less than 1.2*σ*). A loop is identified when two separated train segments along the same chain are bridged by an intermediate segment that is detached from the substrate. A tail corresponds to a terminal segment where only one end is anchored to the substrate via a train, while the remaining beads extend freely into the film interior. These geometric criteria allow an unambiguous assignment of the three adsorption conformations. We investigated the conformation of the adsorbed polymer and found that the constraining effect of the substrate propagates into the film interior through the loosely adsorbed tail conformations. As an indication of the system having reached equilibrium, the total film thickness fluctuated within ±1.5 Å around its mean value of approximately 72.2 Å after 2 ns, and the number of adsorbed beads oscillated within ±3 of its average, with no detectable long-term drift.

**Figure 6:**
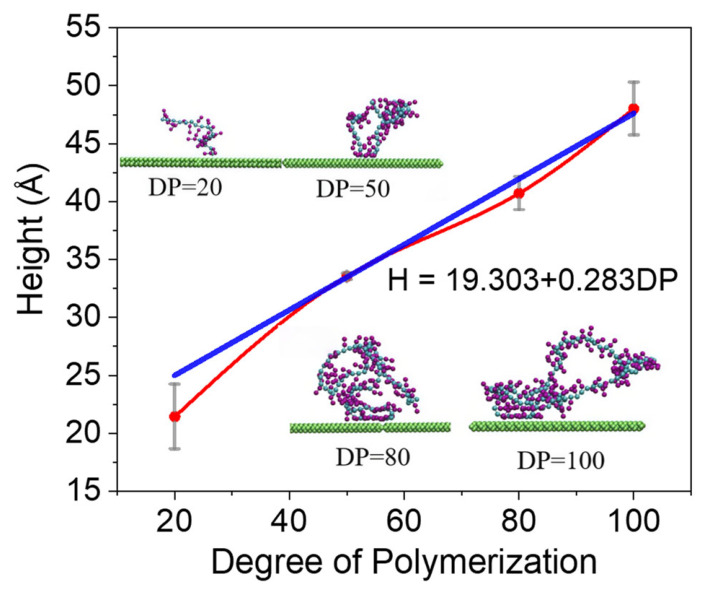
Average penetration height of main chains with different degrees of polymerization (the linear line in the figure is only used for auxiliary observation and does not represent the theoretical scaling relationship).

The relationship between the degree of polymerization and the average penetration height of the main chain is shown in Figure 6. The horizontal axis of Figure 6 represents the degree of polymerization (DP = 20, 50, 80, 100), and the vertical axis shows the average penetration height (in Å) of the adsorbed main chain center of mass in the direction normal to the substrate. The penetration height increases with DP. Longer chains are able to form extended loops and longer tails that protrude deeper into the film interior. It is important to note that the penetration height is an interfacial structural parameter reflecting the degree of chain extension under confinement; it is distinct from the radius of gyration and is not constrained by the Gaussian coil scaling that governs chain dimensions in bulk melts. It is the direct embodiment of segment rearrangement under the condition of limited interface, and it rises with the increase of polymerization degree, which is consistent with the conclusion in the existing literature that the conformation of the adsorbed chain link and tail increases with the chain length [21,22]. The approximate linear trend of penetration height with the increase of DP should be attributed to the joint action of finite film thickness and substrate adsorption site saturation, rather than a universal scaling behavior. With the further increase of polymerization degree or film thickness, this trend is expected to tend toward sub-linear saturation.

According to the experimental and simulation studies of similar P3HT systems, the P3AT main chain behaves as a flexible chain in the bulk phase, and its persistent length is estimated at the order of 1~2 nm [32,34], which is far less than the film thickness of this work. This flexible feature means that the chain has enough bending ability in the vertical direction and can extend to the inside of the film by forming a ring or tail conformation after the substrate is adsorbed, thus supporting the geometric interpretation that the penetration height increases with the increase of the degree of polymerization. The four illustrations in the figure are representative conformations of main chains in films with different degrees of polymerization. Average penetration height refers to the average height of the centroid of the main chain of the adsorption chain in the direction perpendicular to the substrate, indicating the extent to which the chain segment extends into the film. It is a conformational measure under the condition of a limited interface and should be strictly distinguished from the radius of gyration representing the three-dimensional overall size of the chain. Different from the simulation results of polymers without side chains, the penetration height of polymers with long side chains is proportional to the degree of polymerization. On the premise of substrate adsorption, the polymer chain formed three classical adsorption conformations: train, loop, and tail. With the increase of polymerization degree, the chain can form a longer tail or a larger ring, and these chain segments extending outward from the substrate constantly push the center of mass of the main chain to higher places in the film. At the same time, the number of adsorption sites near the substrate is limited. After the train conformation occupies these sites, the redundant segments in the long chain are forced to extend into the film in the form of rings or tails, resulting in a monotonic increase in the penetration height with the chain length [21,22].

In the bulk region of the film, the main chain still maintains the statistical conformation of the Gaussian chain, and its radius of gyration meets the standard scaling relationship that *R*g is proportional to the 1/2 power of *N*. However, the penetration height is a single-particle conformation coordinate defined in the presence of a broken symmetry interface, which measures not the overall size of the chain but the position of the centroid in the constraint direction. Therefore, this quantity is not constrained by the isotropic melt scaling law and its monotonic rise with the increase of chain length is the direct embodiment of the interface effect, rather than a violation of the basic theory of polymers.

**Figure 7:**
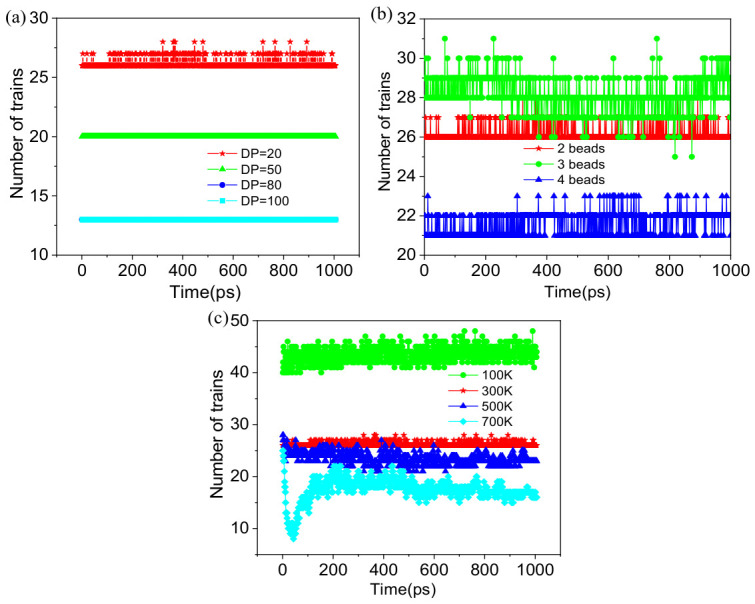
Temporal evolution of the number of trains within 1ns under different conditions: (**a**) different degrees of polymerization (DP = 20, 50, 80, 100), (**b**) different side chain bead numbers (2, 3, 4), and (**c**) different temperatures (100, 300, 500, 700 K).

Different from other studies in ultrathin films of P3AT, the penetration height of the chain can be greater than the propagation depth of the interface effect discussed below and can even reach the phase part of the bulk. The structure of loops and trains can transmit interface effects. The formation of trains occupies adsorption sites on the substrate, thereby directly impeding the formation of loops and tails. In Figure 7, we counted the change in train number during 1ns with different degrees of polymerization, side chain bead numbers and temperatures. Figure 7a depicts the temporal variation of the number of trains with different degrees of polymerization. It is evident that the adsorbent layer with lower degrees of polymerization exhibits a higher number of trains. There is an inverse correlation between the degree of polymerization and the number of trains. Figure 7b shows how the number of trains having three types of side chain beads varies over time. The change trends of the three curves are basically consistent within the statistical fluctuation range, indicating that the influence of side chain length on train formation is not significant. This is physically reasonable because train conformations are primarily determined by backbone-substrate contacts, while side chains do not directly participate in adsorption. Figure 7c illustrates the temporal evolution of the number of trains at different temperatures. It can be observed that lower temperatures correspond to a higher number of trains, while higher temperatures result in a lower number of trains. Furthermore, as the temperature gradually increases, the rate of decrease in the number of trains slows down. Particularly under the conditions of 700 K, the number of trains exhibits significant fluctuations in the initial stage, followed by stabilization. In summary, both the degree of polymerization and temperature have essential effects on the conformational number of trains in the adsorbent layer. Both temperature and degree of polymerization exhibit a negative correlation with the number of trains, while the impact of side chain length on the number of trains is limited. The conformational analysis of tails and loops reveals a lack of apparent regularity in their results. Because the formation conditions for the tail and loop conformations in the adsorption region are comparatively more challenging than those for trains. The number of trains is higher than that of the tail and loop. The conformations of the tail and loop can transmit substrate effects [24,25], but due to the direct contact between the train and the substrate, the thickness of the adsorption region, that is, the dynamic propagation depth from the substrate surface to the disappearance of the stiffness gradient, is significantly less than the thickness of the bulk region. This phenomenon will be discussed in Section 3.2 of our study.

### 3.2. Gradient Stiffness Analysis of Thin Films

#### 3.2.1. Gradient Stiffness of Thin Films with Different Degrees of Polymerization

**Figure 8:**
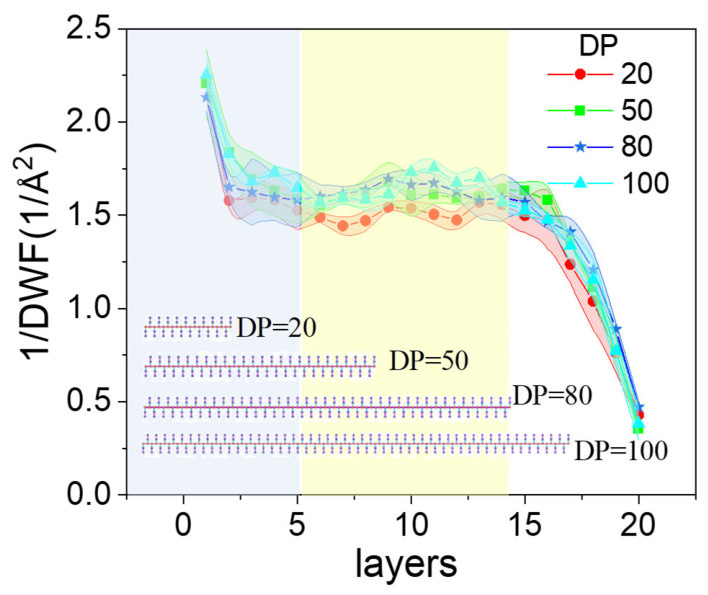
Gradient stiffness of P3AT with different polymerizations along height direction, light blue area is the adsorbed layer region, yellow area is the bulk region and the white area is the free surface region.

The film was evenly divided into 20 layers, with a total thickness of about 72.2 Å, and the exact thickness of each layer was 3.61 Å. The distance between the center of the *n*th layer and the substrate was (*n* − 0.5) × 3.61 Å. Next, we adopted the Debye–Waller factor (DWF) [29] <*u*^2^> to calculate the local molecular stiffness of the P3AT thin film along the height direction, which is a fundamental parameter for predicting the molecular stiffness of polymers in bulk and confined states [29]. All quantities reported in this section were averaged over the equilibrated trajectory beyond the initial 2 ns, ensuring that the stiffness profiles reflect the steady-state behavior of the thin film. The values were calculated by dividing the film into 20 layers, which are parallel to the substrate. DWF quantifies the free volume of molecules and stiffness at picosecond timescale. <*u*^2^> can be obtained from neutron scattering and X-ray. Here, this value is seen as a plateau of mean square displacement (MSD) <*r*^2^(*t*)> at 4 piscoseconds of P3AT coarse-grained beads, corresponding to the localization time scale characterizing the crossover from ballistic to caged subdiffusive regimes for the CG models [29]. The calculation is shown in formula: $\langle r^{2}(t)\rangle =\langle {\left|r_{j}(t)-r_{j}(0)\right|}^{2}\rangle$. The local stiffness is inversely related to the DWF, i.e., 1/<*u*^2^>. Figure 8 shows the distribution of local stiffness in the z-axis direction (height direction) on polymer films with different degrees of polymerization.

The adsorption region is the area close to the substrate with gradually decreasing stiffness, and its thickness corresponds to the measurable influence depth of the substrate on the polymer dynamics. The bulk region is the middle region where the stiffness reaches a relatively stable platform, and the local vibration behavior of the chain in this region no longer changes significantly with the distance from the base. The free surface region is the region where the stiffness decreases rapidly near the vacuum interface. From Figure 8, it can be observed that films formed by P3AT with four different degrees of polymerization exhibit similar behavior. We divide the film into three regions. The first region corresponds to the light blue area in the graph, referred to as the adsorbed layer region. The second region corresponds to the yellow area in the graph, known as the bulk region. The third region corresponds to the white area in the graph, referred to as the free surface region. The film layers close to the substrate (within 3.612 angstroms) show the highest stiffness. Around the thickness of the fifth layer (18.062 angstroms), the curve becomes parallel to the horizontal axis, indicating a transition of the polymer from an adsorbed layer to a more bulk-like nature. This fifth layer can be considered the dynamic spreading depth due to substrate effects. In contrast to reference [21], stiffness within the dynamic spreading depth does not exhibit a linear increment, which is attributed to varied adsorption conformations at the interface. The conformation of adsorbed chains at the interface has a restraining effect on polymer stiffness.

The local stiffness in this paper is measured by the reciprocal of the Debye–Waller factor, which reflects the vibration amplitude of a single bead constrained by the transient cage formed by the surrounding beads on a time scale of about 4 ps. It belongs to the local fast scale property and is directly related to the local packing density and the curvature of the L-J potential, rather than the characterization of the overall flexibility of the chain. The duration length represents the exponential decay correlation distance along the chain skeleton direction, which belongs to large-scale chain statistics. At the coarsening level, the local cage effect of the monomer is mainly determined by non-bond contact. Because the non-bond parameters of main chain beads and side chain beads in our model are the same or very close, it is in line with physical intuition that the local vibration environment is insensitive to the chain length and side chain length. We clearly write that the insensitivity of local stiffness to chain length and side chain length does not mean that the duration length does not change but reflects the essential difference between the picosecond cage effect and large-scale chain statistics [29].

In addition, near the free surface, the stiffness can be seen as a slow reduction process because in this region the influence of the substrate on the polymer has completely disappeared and each bead has more freedom, resulting in a reduction in stiffness. The stiffness variation near the free surface and the stiffness of the adsorption area do not exhibit rotational symmetry, which is significantly different from the results of the free-standing thin film in reference [29]. Combining Figure 6 and Figure 8, the following conclusion can be drawn. Ultra-thin films adsorbed onto substrates, in general, cannot be removed using a suitable solvent wash. The reason for this is that within the film, the polymer chains adsorbed at the interface can hinder their extension into the bulk phase. Within this thickness range, the polymer chains play a role in connecting the interface and the free surface, effectively enhancing the adhesive strength of the film. As a result, the film cannot be washed away by a good solvent. The tri-regime stratification identified here from the stiffness profile adsorbed, bulk-like, and free surface regions is an operational definition derived directly from the simulation data. Although direct experimental validation of this stiffness-based layering remains challenging at the single-nanometer scale, indirect experimental support exists. Tian et al. [22] reported that in metastable polymer adsorption layers, suppressed interfacial dynamics propagate into the film interior, creating a dynamical gradient zone between the substrate and the bulk. Zuo et al. [25] further demonstrated that the conformation of chains adsorbed at the interface, flattened trains and extended loops, mediates this propagation. These experimental observations are qualitatively consistent with the adsorbed and bulk regions delineated by our stiffness analysis. Direct experimental verification of the predicted stiffness gradient could be pursued in future studies using interface-sensitive techniques such as sum-frequency generation vibrational spectroscopy or X-ray reflectivity.

#### 3.2.2. Gradient Stiffness of Thin Films with Different Side Chain Beads Number

**Figure 9:**
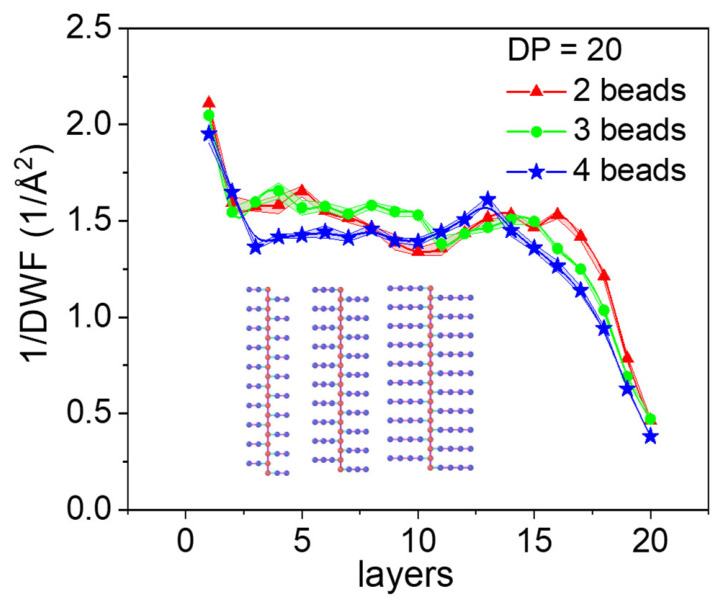
Gradient stiffness of P3AT thin film with different side chain bead numbers.

Figure 9 presents the stiffness (1/DWF) variation graphs for P3AT with different side chain lengths. P3ATs with varying side chain lengths have the same polymerization degree of 20 at 300 K. Thin films of P3ATs with side chain lengths of 2 beads and 3 beads exhibit similar trends. The depth of substrate influence on the film extends up to 5 layers, while the bulk phase thickness can reach up to 15 layers. In the case of P3AT with a side chain length of 4 beads, the substrate’s influence depth reduces to three layers, and the bulk phase advances. Furthermore, compared to the cases with 2 or 3 beads in side chains, the height of the bulk phase in P3AT with 4 beads is shorter. The bulk phase thickness of the P3AT film with side chains of 4 beads is lower than that with 2 or 3 beads. Compared to side chains with 2 or 3 beads, the P3AT film with 4 beads exhibits a slightly thinner adsorbed region and a somewhat thicker free surface region; however, these differences are comparable to the statistical fluctuations inherent in the current simulations. While longer side chains may influence the spatial extent of interfacial and free-surface zones, a definitive conclusion would require extended sampling or multiple independent replicas. These results are consistent with the trend in average extension height. Based on block-averaged estimates, the plateau stiffness values in the bulk region fluctuate within ±0.08–0.10 Å^−2^ for the three side chain lengths studied. The differences in the mean plateau stiffness among the 2-bead, 3-bead, and 4-bead systems are on the order of 0.03–0.07 Å^−2^, which fall within the range of temporal fluctuations. These differences are within statistical uncertainty and thus no definitive conclusion can be drawn. For P3ATs, having longer side chains leads to a reduction in the thickness of both the adsorbed layer and the bulk layer, while the thickness of the free surface region increases. In the process of designing an interface system, the modulation of interfaces can be achieved by controlling the length of side chains.

#### 3.2.3. Gradient Stiffness of Thin Films at Different Temperatures

**Figure 10:**
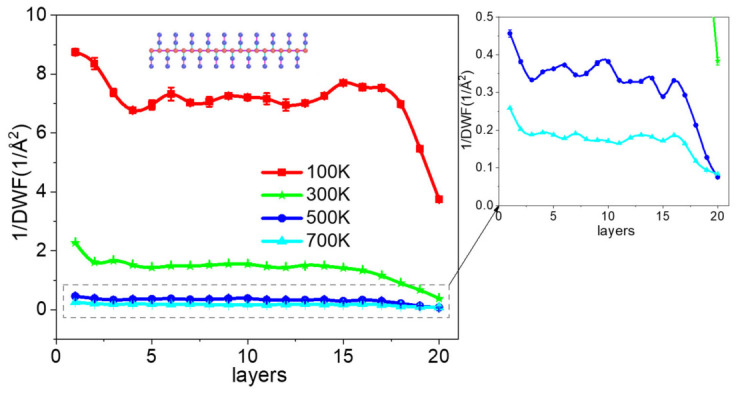
P3AT thin film stiffnesses at different temperatures. The polymer P3AT’s polymerization is 20, side chain has 2 beads, the illustration provides enlarged views of 500 K and 700 K curves to reveal the weak stiffness changes near the free surface, which are not easy to distinguish on the scale of the main diagram. The data shown in the illustration is completely consistent with that in the main figure, and only the vertical axis scale is enlarged.

As shown in Figure 10, the schematic illustration depicts the stiffness distribution of P3AT thin films on a substrate at different temperatures. At 500 K and above, the stiffness value is low, and its layer-by-layer change is almost imperceptible in the main figure. The function of the illustration is to magnify these trends without changing the basic data. The simulations for all four temperatures were conducted with a polymerization degree of 20 and a side chain length equivalent to 2 beads of P3AT. Similar to the two cases mentioned above, the gradient change in stiffness can be divided into three regions: the adsorption region, the bulk region, and the free surface region. From a trend perspective, under different temperatures, the four types of stiffness of the thin film on the substrate exhibit a similar phenomenon. The stiffness values of the thin film initially decrease, then reach a plateau around the fourth or fifth layer, maintaining a relatively constant value. Subsequently, at approximately the 15th layer height, the stiffness values experience a rapid drop. The curve for 100 K temperature displays the highest stiffness value, whereas at a 700 K temperature, the stiffness value is the lowest. Thus, numerically, regardless of the region, higher temperatures correspond to lower stiffness values. In terms of the rate of stiffness variation, with increasing temperature, the stiffness reduction becomes more pronounced. Based on the findings of Wang et al. [34], it is known that the glass transition temperature (*T*_g_) of P3AT is below 300K. In the presented simulations, P3AT at 100K is in a glassy state. At temperatures of 500K and higher, P3AT has transitioned into a viscoelastic state. Consequently, the abrupt decrease in stiffness values can be attributed to this transition.

### 3.3. Phonon Modes Analysis of Thin Film and Similarity Analysis Between Side Chain and Backbone Chain

The frequency of phonon density of state (PDOS) is a useful way to understand the vibrational modes per unit frequency and explain the substrate effect on polymer thin film. All PDOS calculations and similarity analyses were based on the equilibrated trajectory (*t* > 2 ns) to exclude any transient effects from the initial relaxation phase. Having verified the equilibration of the thin-film systems and established the gradient stiffness characteristics in the preceding sections, we now turn to the vibrational dynamics. The phonon density of states was computed from the velocity autocorrelation function over the equilibrated trajectory, providing spectral information on the molecular-level vibrational modes that complements the stiffness analysis.

We calculated the normalized PDOS, which was applied by fast Fourier transformation of the velocity autocorrelation function. The PDOS formula is shown as:

(1) $$PDOS(\omega )=\int \frac{\sum_{i=1}^{N}v_{i}(0)\cdot v_{i}(t)}{\sum_{i=1}^{N}v_{i}(0)\cdot v_{i}(0)}\cdot e^{\left(-2\pi iwt\right)}dt$$

where *i* is the imaginary unit, *N* is the total number of particles, *v_i_*(0) is the initial time velocity, *ω* is the phonon frequency, and *v_i_*(*t*) and are velocities of the *i*th bead at time *t* [43].

**Figure 11:**
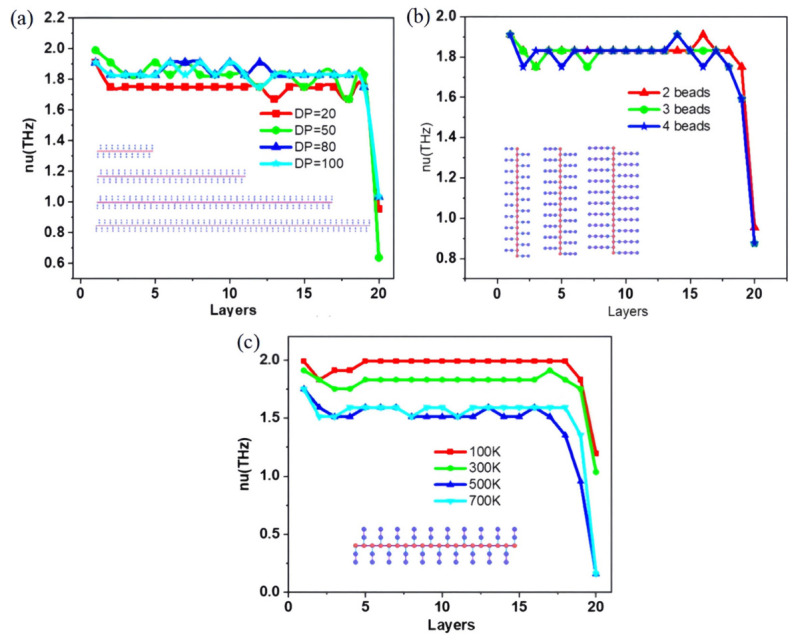
Phonon density of state analysis of P3AT at different degree of polymerization (**a**), side chain beads number (**b**) and temperatures (**c**).

Figure 11 illustrates the relationship between phonon modes and the number of layers in the thin film. To capture the phonon frequency status of beads within each layer, we extracted representative frequencies from the phonon density of states. The results are presented in the Figure 11. The characteristic frequency plotted in Figure 11 is the peak frequency of the PDOS, the frequency at which the vibrational density of states reaches its maximum. This peak frequency reflects the most dominant vibrational mode of the beads in that layer. Figure 11a depicts the relationship between phonon vibration frequency and the number of layers for different degrees of polymerization. Figure 11b illustrates the relationship between phonon vibration frequency and the number of layers for various numbers of side chain beads, while Figure 11c shows the relationship between phonon vibration frequency and the number of layers at different temperatures. From the trends of phonon frequencies with respect to the number of layers in the three plots, it can be observed that, despite differing external conditions, they exhibit a consistent pattern. This trend bears resemblance to the stiffness gradient variations in the thin film. Similarly, the phonon frequencies can also be categorized into three regions: the adsorption region, the bulk region, and the free surface region. Moving from the adsorption region to the bulk region, there is a noticeable decrease in phonon frequencies. The phonon frequencies in the bulk region remain relatively constant. In the free surface region, phonon frequencies experience a rapid decline.

From Figure 11a–c, it is evident that the P3AT with a degree of polymerization of 20 exhibits the lowest phonon frequency. Phonon frequencies are slightly lower for the shortest chains (DP = 20) compared to longer chains, but no statistically significant difference is observed among DP = 50, 80, and 100. This suggests that beyond a certain chain length, the vibrational dynamics become essentially independent of the degree of polymerization. The increase in side chain length does not significantly affect phonon frequencies, while temperature has a notable impact on them. From Figure 11c, it can be observed that there is an inverse relationship between phonon frequency and temperature. Phonon frequencies at low temperatures are relatively higher compared to those at high temperatures. Furthermore, when the temperature surpasses a certain threshold, phonon frequencies cease to exhibit significant changes. Conducting a layered investigation into the phonon frequencies of polymers will significantly enhance our understanding of polymer structure and the vibrational state of adsorbed particles at the molecular scale. It is worth noting that the spatial variation of phonon frequencies closely mirrors the gradient stiffness profile established in Section 3.2. Specifically, the adsorbed region exhibits the highest phonon frequencies, corresponding to the elevated stiffness near the substrate; the bulk region displays relatively constant frequencies, consistent with the stiffness plateau; and the free surface region shows a rapid decline in frequency, matching the sharp stiffness reduction at the vacuum interface. This consistency between two independent measures of stiffness, derived from the Debye–Waller factor at picosecond timescales and vibrational frequencies extracted from the velocity autocorrelation function, provides strong cross-validation of the tri-regime gradient picture and reinforces the reliability of the present phonon analysis.

**Figure 12:**
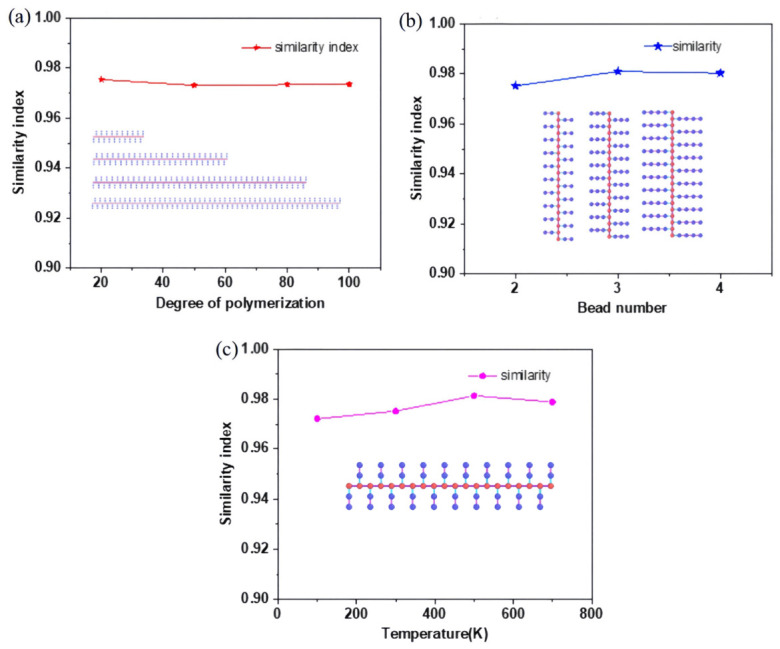
Similarity index of P3AT at different conditions: (**a**) different degree of polymerization, (**b**) different side chain beads number, (**c**) different temperatures.

Figure 12a illustrates the similarity index [44,45,46] of P3AT at various degrees of aggregation. All four models utilize two side chain beads and a temperature of 300 K. From the figure, it can be observed that despite varying degrees of aggregation, there is a high degree of similarity, approximately 98%, between the phonon modes of bead 1 in the main chain and bead 2 in the side chain. Similarly, from Figure 12b, it can be deduced that the number of side chain beads has a minor impact on the similarity index between the two types of beads. Figure 12c displays the influence of temperature on the similarity index. As the temperature increases, the similarity index experiences a slight rise. After reaching a certain temperature threshold, the coefficient stabilizes and may even exhibit a modest reduction. Considering the influences of these three factors, it can be concluded that neither the degree of polymerization nor the number of side chain beads affects the similarity index between the main chain and side chain. Temperature has a relatively minor impact on the similarity index.

### 3.4. Energy Decomposition

**Figure 13:**
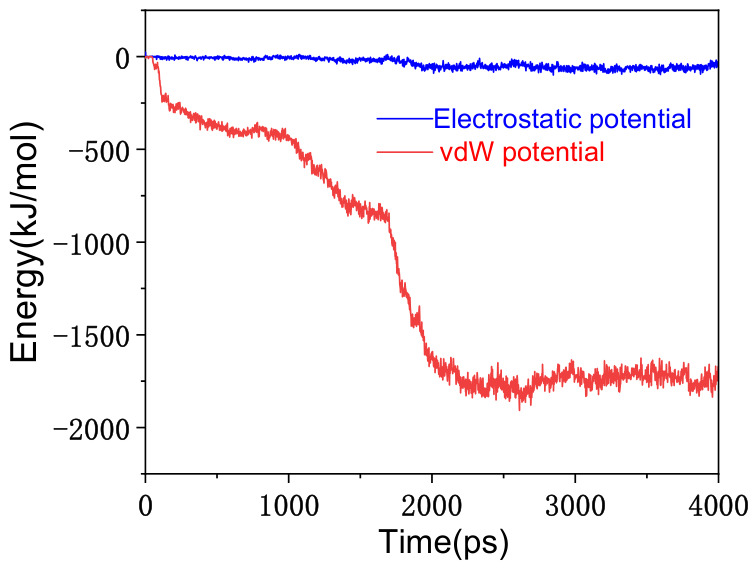
Evolution of electrostatic potential and vdW potential during simulation.

In the view of Tian et al. [22], the propagation of suppressed dynamics originates from the interface. To gain a deeper understanding of the nature and impact of interfacial interaction force, we employed an energy decomposition [47] approach to investigate the constituents and the contribution of each component to the overall interaction force. In this section, we performed a force field-based energy decomposition to comprehend the nature of interfacial interactions from the perspective of weak intra-molecular interactions. The analysis was conducted using the Multiwfn [48] software. Non-bonded interactions within the molecular force field can be categorized into electrostatic and van der Waals interactions, further subdivided into repulsion, generating repulsive forces, and dispersion, providing attractive forces. In this portion, we utilized a full atomic model and quantum chemical computations for our investigation. We first examined the temporal evolution of these two interactions. The outcomes are depicted in Figure 13. It should be noted that Figure 13 displays the time evolution of the electrostatic and van der Waals potentials over the full simulation, including the initial equilibration phase. The drift observed during the first 2 ns corresponds to the relaxation of the system from its initial configuration toward equilibrium. The stable values attained thereafter confirm that the system is well equilibrated for the subsequent energy decomposition analysis. The quantitative equilibrium interaction energy components, obtained by averaging over the production portion of the trajectory (*t* > 2 ns), are reported separately in Table 1. Because the early-time values in Figure 13 are influenced by the initial transient relaxation, they should not be directly compared with the equilibrated averages in Table 1. Table 1 serves as the definitive quantitative reference for the interfacial interaction energetics discussed in this work.

In Figure 13, the electrostatic potential remains nearly constant throughout the simulation, and its values are mainly close to zero. In contrast, the absolute value of the van der Waals potential gradually increases initially and then experiences a sharp change around 2 ns, eventually stabilizing around 1700 kJ/mol. A clear conclusion can be drawn: the electrostatic interaction in interfacial interactions is significantly smaller than the van der Waals interaction. Next, we conducted a more detailed decomposition of the van der Waals interactions. The energy decomposition results are presented in Table 1. Table 1 presents the energy decomposition results obtained through calculations using Multiwfn. The magnitude of the dispersion interaction value (−5792.46) is significantly larger than that of the electrostatic interaction. The value of repulsion accounts for 36.73% of the absolute value of dispersion. From the table, it is evident that the fundamental nature of interfacial interactions is primarily governed by the dispersion effect, with the electrostatic contribution playing a minor role, possibly even negligible. Additionally, the repulsion effect substantially counteracts the attractive influences of both dispersion and electrostatic interactions, exerting a counteractive role in the interfacial attraction.

**Figure 14:**
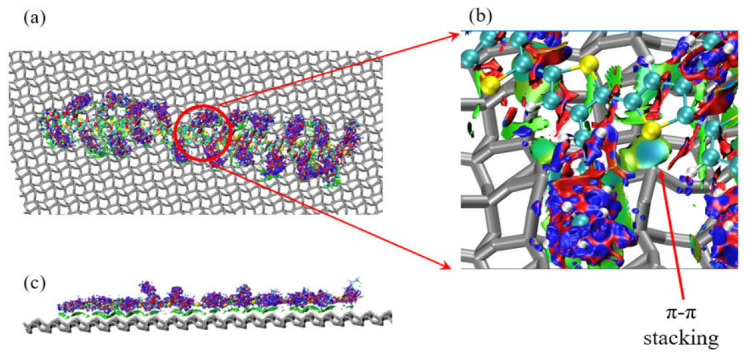
Interfacial interaction between P3AT and silica. (**a**,**b**) Top views of the aNCI isosurfaces for an adsorbed P3AT chain on the silica substrate; (**c**) side view of the same system. The blue color represents strong attraction, the green color represents van der Waals interaction and the red color represents strong repulsion.

Finally, we employed the aNCI (analytical non-additive correction of interaction) method to visualize the spatial distribution of non-covalent interactions. In the aNCI scheme, the color coding reflects the sign and magnitude of sign(λ_2_)ρ: blue indicates strongly attractive interactions (significantly negative sign(λ_2_)ρ, typically associated with hydrogen bonds or oriented π–π stacking), green corresponds to weakly attractive van der Waals dispersion interactions, and red indicates steric repulsion (positive sign(λ_2_)ρ). The outcomes are presented in Figure 14. Figure 14a,b show the top views of the aNCI isosurfaces for an adsorbed P3AT chain on the silica substrate. Distinct blue isosurfaces can be observed between the thiophene rings of the main chain and the silica surface. These blue regions originate from O–H⋯π and Si–O⋯π interactions between the delocalized π-electrons of the thiophene rings and the silanol (Si–OH) groups on the silica surface. Such interactions are directional and locally enhanced, giving rise to a strong attractive signature in the aNCI analysis. The green isosurfaces, which are pervasive throughout the interfacial region, arise from non-specific dispersion interactions between the alkyl side chains and the substrate, as well as among polymer segments.

Figure 14c shows a side view of the same system. A broad green region is evident between the polymer and the substrate, representing the dominant van der Waals dispersion interactions that constitute the bulk of the interfacial adhesion. Although the green region appears concentrated near the substrate in this cross-section, this is because the slice intersects the direct polymer-substrate contact zone, where the electron density overlap generates a detectable but weak attraction signal. In the top views (Figure 14a,b), the green isosurfaces are seen to extend broadly around the thiophene rings and side chains, confirming that dispersion interactions are not localized but permeate the entire interface. This spatial picture is fully consistent with the energy decomposition results in Table 1, where the dispersion contribution overwhelmingly dominates over the electrostatic contribution. The green sea in Figure 14 is thus the visual manifestation of the dispersion-dominated interfacial energetics, while the localized blue patches represent the additional specific enhancement from π-mediated interactions with surface silanol groups. Together, these two types of interactions, pervasive dispersion and localized specific attraction, cooperatively stabilize the adsorption of P3AT on silica.

## 4. Conclusions

This molecular dynamics study elucidates the gradient stiffness and vibrational characteristics of poly(3-alkylthiophene) (P3AT) thin films supported on a silica substrate. Stiffness, quantified via the inverse of the Debye–Waller factor derived from mean square displacement at 4 ps, was profiled across 20 stratified layers of the film. The stiffness–depth relationship consistently exhibits a tri-regional trend across all tested systems: the adsorbed region shows a gradual decline, the bulk region remains largely constant, and the free surface region undergoes a sharp decrease. Polymerization degree was found to have negligible impact on stiffness distribution. Similarly, side chain lengths of two or three beads yield analogous stiffness profiles. For the four-bead side chain, a slight tendency toward a thickened free surface region and a reduced bulk region is observed, but the differences are comparable to the statistical fluctuations of the current simulations and require further verification. Temperature markedly influences stiffness, with a one-order-of-magnitude reduction observed above the glass transition temperature. Phonon mode analysis reveals an inverse correlation between polymerization degree and vibrational frequency, while side chain length exerts minimal influence. Temperature also negatively correlates with phonon frequency. A high phonon mode similarity index between the main and side chains indicates strongly coupled vibrational dynamics. Interfacial energy decomposition confirms the dominance of van der Waals interactions, with a distinct contribution from π–π stacking between P3AT and the substrate. These findings provide molecular-scale insights into the gradient mechanical properties, vibrational behavior, and interfacial interactions of P3AT thin films, offering valuable guidance for the rational design of polymer-based electronic devices. It should be noted that the present uncertainty estimates are based on block averaging of single production trajectories. Extended sampling and independent replica simulations would be valuable for refining the confidence intervals on the reported trends, particularly for the subtle variations in regional thicknesses induced by side chain length changes. The present findings are specific to the P3AT/silica interface. Whether similar stiffness gradients and vibrational coupling phenomena persist on other technologically relevant substrates, such as graphite, gold, or ITO, remains an open question that warrants dedicated future investigation. In addition, the persistent length is the core parameter to characterize the chain rigidity, which is of great value for understanding the relationship between the film thickness and the chain conformation. In the follow-up study, a periodic boundary simulation without base conditions will be specially designed, and the long chain system will be fully sampled. The persistent length under different side chain lengths will be calculated systematically, and then its correlation with local stiffness and phonon modes will be discussed in depth.

## Figures and Tables

**Table 1:** Interaction energy components.

Interaction	Electrostatic	Repulsion	Dispersion	Total
Value (kJ/mol)	−82.41	2127.64	−5792.46	−3727.23

## Data Availability

The original contributions presented in this study are included in the article. Further inquiries can be directed to the corresponding author.
